# Ultrasound-Guided Stellate Ganglion Block With Preserved Motor Function for Upper Extremity Surgery

**DOI:** 10.7759/cureus.18537

**Published:** 2021-10-06

**Authors:** David H Cho, Jichang Li, Andrui Nazarian

**Affiliations:** 1 Anesthesiology, Harbor University of California Los Angeles Medical Center, Torrance, USA

**Keywords:** stellate ganglion block, regional anesthesia, acute pain management, anesthesiology, hand surgery

## Abstract

While stellate ganglion blockade (SGB) is commonly used in the treatment and management of patients who suffer from chronic pain, we are reporting a case where an ultrasound-guided SGB was used for management of acute perioperative pain for a patient undergoing upper extremity surgery. The patient was classified as the American Society of Anesthesiologists (ASA) class 1, without any significant past medical history, including no history of chronic pain, opioid use, or peripheral neuropathy. The patient was scheduled for tendon repair of the hand following trauma with subsequent lacerations. While general anesthesia, a brachial plexus blockade, or combination of the two are generally used in current practice for upper extremity surgery, these typically do not allow for intraoperative evaluation of motor function. In our case, an ultrasound-guided SGB was used to provide analgesia while still allowing for intraoperative assessment of motor function during the critical components of the repair. This case illustrates the potential advantages of an ultrasound-guided SGB for decreasing acute postoperative pain scores, decreasing overall postoperative pain medication use, as well as maintaining intraoperative motor function in cases where such monitoring may be advantageous and allow for optimal surgical repair.

## Introduction

When performed correctly, brachial plexus blockade can abolish both sensory and motor function to the upper extremity, providing good intraoperative and postoperative analgesia [1-2]. However, these blockades can be cumbersome for patients as they no longer can move their extremities.

There have been limited studies demonstrating the effectiveness of acute postoperative pain management following a stellate ganglion blockade (SGB) in upper extremity surgeries. The stellate ganglion provides sympathetic innervation to the upper extremity, head, and neck, and can be selectively blocked without effects on the motor function to these regions. Literature supports the use of SGB for chronic pain which is sympathetically mediated, such as in Complex Regional Pain Syndromes, however, the potential for the sympathetic nervous system to contribute to acute nociceptive pain has not been investigated in detail [3-4]. It is believed that the somatic nervous system is involved in acute nociceptive pain, and this is the current target of perioperative analgesia, however, there have been a select number of case reports in patients with upper extremity surgery that showed improvements in postoperative pain scores and opioid use in the setting of an SGB, indicating that perhaps trauma, surgical stress, and inflammation may trigger sympathetic nervous system activation [5-8]. An SGB provides an additional advantage over conventional nerve blockade of not producing motor blockade, allowing the surgeon to assess motor function of the operated arm in the immediate postoperative period.

Here we report a case that looks at intraoperative patient participation, postoperative pain scores, and perioperative analgesic requirements with the use of ultrasound-guided SGB in a patient undergoing upper extremity surgery. Our report continues to demonstrate a technique that can potentially minimize acute perioperative pain with preservation of motor function to the limb as well as decreasing postoperative analgesic use. We hope that it will provide more evidence for the use of the SGB for management of acute perioperative pain.

## Case presentation

The SGB in our case was performed in the preoperative area where the patients’ blood pressure, heart rate, and oxygen saturation were monitored throughout the procedure. The patient was in supine position with the head slightly extended and rotated contralateral to the block side. The overlying skin was prepped with a chlorhexidine solution and draped in a sterile manner. Ultrasound was used to identify the cricoid cartilage, the carotid sheath laterally, and deep to these structures, the transverse process of C6 or Chassignac’s tubercle. Guided by real-time ultrasound imaging, a Stimuplex 21-gauge needle (Havel's: Cincinnati, OH) was advanced in an in-plane technique until the needle tip was inside the longus colli muscle where the local anesthetic was administered. The blockade was confirmed by the presence of a Horner’s syndrome in patient as well as an increase in temperature in the ipsilateral extremity with liquid crystallography thermometers. Additionally, the neurologic evaluation revealed no sensory or motor loss in the ipsilateral upper extremity.

Case 1: A 24-year-old man was evaluated at the hospital for tendon lacerations from a knife on his left hand and was scheduled for a left-hand tendon repair. The patient was otherwise healthy without any significant history including no history of chronic pain syndrome, opioid use, or peripheral neuropathy. The patient was consented for and received a preoperative ultrasound-guided SGB with 20 ml of 0.5% ropivacaine. A 21 gage, 2 inch echogenic needle (Havel's 2 inch 21 guage stimuplex needle), was used to give an injection into the longus coli muscle at approximately the C6 level using ultrasound guidance. Immediately following the blockade, the patient reported a pain score reduction from 7 to 1 on the visual analog scale (VAS). Neurologic evaluation revealed intact sensation and motor function. The patient then was taken to the operating room where he received supplemental local anesthetic administration by the surgical team consisting of 5 ml 1% lidocaine with 5 ml 0.25% bupivacaine. During the surgical procedure, the patient was asked to participate with motor movements during the critical components of the procedure. Once satisfactory participation was achieved the patient underwent general anesthesia which was induced with 2 mg/kg propofol IV bolus, maintained with propofol infusion at a rate of 150 mcg/kg/min, and a laryngeal mask airway was inserted for assisting ventilation. The total tourniquet time for this case was 1 hour and 35 minutes all of which was prior to the general anesthesia, when the patient was awake.

Postoperatively, the patient reported a pain score of 0 with no pain medication administration within the first hour upon arrival to the post anesthesia care unit (PACU). During the following 3 hours, the patient had an increased pain level of 7, and received 0.4 mg Dilaudid followed by 1 tablet of norco 10 mg/325 mg right before being discharged home from the hospital. The patient had no motor blockade and the surgical team was able to reassess motor function postoperatively in the recovery area.

## Discussion

Brachial plexus blockade has become an integral component of the management of acute perioperative pain in upper extremity surgeries as it has consistently provided lower pain scores and a reduction in the consumption of opiates postoperatively, as well as improved perioperative hemodynamics and decreased opioid-related side effects [1-2]. Brachial plexus blockade, however, inhibits motor functions to the upper limb.

The use of SGB for chronic pain syndromes has been well established in the literature. SGBs have been successfully used in chronic pain associated with complex regional pain syndrome (CRPS), vascular insufficiency, and post-herpetic neuralgia. The SGB is unique from a brachial plexus blockade, in that it targets the sympathetic innervation to the upper limb, head, and neck without affecting sensory and motor functions [3-4]. While the literature has shown the success of blocking the sympathetic nervous system in chronic pain, there are only a handful of studies that report its role in acute nociceptive pain in the perioperative phase.

Recent studies have suggested the involvement of the autonomic nervous system in the pathogenesis of acute pain. This may be a result of the activation of the sympathetic nervous system during times of stress such as pain. Furthermore, in certain situations, the autonomic nervous system can amplify pain rather than suppress it [5-6]. Acute nociceptive pain is not entirely dependent on peripheral nociceptors; however, it contains neuroendocrine and autonomic components as well [5-6]. Acute inflammation is in part dictated by the sympathetic nervous system through its influence on the arteriolar tone. In upper extremity surgery, the vasodilatory effects of the SGB potentially allow for the washout of the inflammatory mediators in the surgical arm which could be contributing to sensitization of nociceptors [8].

An SGB for acute postoperative upper extremity pain was successfully performed by Kakazu et al. which resulted in a significant reduction in postoperative pain from a 10 to 0 following open reduction and internal fixation of a humeral fracture [9]. Further, McDonnell et al. reported that the SGB has the potential to reduce postoperative pain scores, opiate consumption, and opiate-related side effects in patients undergoing open reduction and fixation of upper extremity fractures. Subsequently, in a randomized, double-blinded, placebo-controlled study, Kumar et al. demonstrated lower pain VAS scores as well as decreased tramadol patient-controlled analgesia use [8]. These studies have just begun to explore the use of preoperative stellate ganglion blocks in the management of acute postoperative pain, thus demonstrating the role of the sympathetic nervous system in acute pain. In contradiction to the aforementioned studies, however, Choi et al. conducted a randomized, blind, controlled clinical trial which led to the conclusion that there was no difference between the SGB and control groups in terms of VAS pain scores, vital signs, and analgesic requirements in the first 48 hours following arthroscopic shoulder surgery [10].

We review a case report where SGB was performed for intraoperative and postoperative use in tendon repair of the hand. It is important to recognize that the patient noted significant reductions in pain immediately following the SGBs. In addition, they were able to better tolerate tourniquet pain during the duration of the awake period of the surgery. Of particular interest was that the patient was able to participate during the critical components of the surgery by moving his digits voluntarily. This additional participation allowed for optimal surgical repair and eventual recovery. Further, the patient had low pain scores at 1 and 3 hours postoperatively and required lower doses of parenteral and PO opioids in comparison to patients who had no regional anesthetic technique used. While looking at other similar hand tendon repair surgeries which were done at our institution under general anesthesia or supraclavicular blocks, the obvious intraoperative patient interaction was lost, and with general anesthesia, the patients had higher 1- and 3-hour postoperative pain scores and received higher total amounts of perioperative narcotics than the SGB patients. The supraclavicular block patients may have had better pain scores and less analgesics postoperatively, however, the patient participation component could not be accomplished with this block as the motor function is completely abolished. While the supplemental intraoperative local anesthetic may have aided in achieving adequate analgesia, it seems as if the SGB worked synergistically to allow for surgical analgesia, perhaps which neither could have done alone.

## Conclusions

In conclusion, these cases illustrate the potential advantages of ultrasound-guided SGB for decreasing acute postoperative pain scores, decreasing overall pain medication use, as well as allowing for perioperative mobility of the extremity, therefore, allowing the surgeon to monitor intraoperative as well as the postoperative motor function of the upper extremity in cases where a motor function evaluation may be advantageous. While an SGB may not be the ideal choice for every upper extremity surgery, it can be a great tool in an anesthesiologist’s toolbox in specific upper extremity cases which require patient participation in the surgery where the SGB can provide some level of analgesia while still allowing for the patient to have motor function for intraoperative evaluation. It can also be used in cases where close postoperative neurologic evaluation is warranted and therefore a brachial plexus block cannot be used. In these cases, an SGB can be considered postoperatively for reduction of acute postoperative pain without loss of motor function. Nevertheless, larger prospective studies with controlled variables are necessary to further evaluate the role of SGBs in acute nociceptive pain management. The limitations of presenting a case report are the number of subjects studied and hence no true comparisons can be made, but only observations based on these cases can be reported. However, this case report can be drawn upon for prospective studies for further analysis, as well as provide further evidence for the use of stellate ganglion blocks.
